# Safety and efficacy of carbon dioxide laser therapy for vocal cord leukoplakia: A systematic review and meta-analysis

**DOI:** 10.1097/MD.0000000000041539

**Published:** 2025-02-14

**Authors:** Jiao Liang, Yanan Zhou, Zhoujie Xu, Xingwei Cao

**Affiliations:** a College of Traditional Chinese Medicine, Chongqing Three Gorges Medical College, Chongqing, China; b Department of ENT, Hospital of Traditional Chinese Medicine of Emeishan City, Leshan, Sichuan Province, China; c Department of Spinal Surgery, Hospital of Traditional Chinese Medicine of Emeishan City, Leshan, Sichuan Province, China.

**Keywords:** CO_2_ laser, meta-analysis, vocal cord leukoplakia

## Abstract

**Background:**

Vocal cord leukoplakia is a precancerous lesion for which early surgical intervention following ineffective conservative treatment is the predominant approach. Carbon dioxide (CO_2_) laser treatment is a novel, minimally invasive therapy. However, there is insufficient evidence to suggest that CO_2_ laser treatment is superior to conventional surgical methods in terms of efficacy and safety. Therefore, this meta-analysis aimed to evaluate the efficacy and safety of CO_2_ laser treatment vs conventional surgical treatment for vocal cord leukoplakia.

**Methods:**

A literature search was conducted using Preferred Reporting Items for Systematic Reviews and Meta-Analyses guidelines across 8 databases. Studies on the use of carbon dioxide laser therapy for vocal cord leukoplakia were included in the review. Two researchers conducted a systematic search for randomized controlled trials in PubMed, Embase, Cochrane, Web of Science, China National Knowledge Infrastructure, Wanfang, Chinese Science and Technology Periodical Database, and Chinese Biomedical Literature databases. Meta-analysis was performed using Stata 18.0, and RevMan 5.2 software.

**Results:**

The findings indicated that Compared with conventional surgery, CO_2_ laser surgery significantly reduced the recurrence rate of vocal cord leukoplakia (odds ratio [OR] = 0.25, 95% confidence interval, CI [0.12, 0.51]). CO_2_ laser surgery significantly improved the cure rate of vocal cord leukoplakia (OR = 3.07, 95% CI [1.17, 8.09]). Voice quality following CO_2_ laser surgery was significantly superior to that following conventional surgery, particularly in terms of fundamental frequency disturbance (standardized mean difference [SMD] = −0.8, 95% CI [−1.25, −0.34]) and amplitude disturbance (SMD = −0.52, 95% CI [−0.95, −0.09]). The CO_2_ laser group exhibited a lower incidence of postoperative adverse reactions (OR = 0.32, 95% CI [0.03, 3.19]); however, this difference was not statistically significant.

**Conclusion:**

The results of this meta-analysis suggest that CO_2_ laser surgery can reduce the recurrence rate of vocal cord leukoplakia, improve the cure rate, and enhance voice quality. Therefore, CO_2_ laser surgery is recommended as a minimally invasive surgical approach for vocal cord leukoplakia.

## 1. Introduction

Vocal cord leukoplakia, also known as laryngeal leukoplakia, is a type of precancerous lesion characterized by hoarseness as the initial symptom, primarily manifested as fluctuating hoarseness, throat irritation, sore throat, and/or chronic cough. There is limited epidemiological data available.^[1–3]^ Bouquot et al conducted a statistical analysis on the incidence rate of clinically diagnosed laryngeal leukoplakia among the population of Rochester in the United States from 1934 to 1984. The incidence rates for males and females were 10.2 per 100,000 and 2.1 per 100,000, respectively.^[4]^ The morphological of vocal leukoplakia manifestations exhibit variability, with lesions confined to the anterior portion of the vocal cords or extending throughout their entire length. These manifestations may present as diffuse growth in patches or as white thickened mucosa with distinct boundaries, accompanied by irregular external protrusions. Some lesions may also manifest as ulcerative formations or in combination with erythema. The incidence rate of vocal cord leukoplakia is complex, and the primary etiological factors may be closely associated with smoking, alcoholism, viral infection, inhalation of irritant substances, voice damage, and laryngopharyngeal reflux.^[1,2,5]^

Different pathological subtypes exhibit varying prognoses, and the treatment approach emphasizes early detection, diagnosis, and intervention to prevent the progression of vocal cord leukoplakia and potential malignant transformation. Treatment modalities include conservative management and surgical resection. Conservative treatment primarily focuses on voice rest, avoidance of inflammatory stimuli, and cessation of smoking and alcohol consumption. For cases of suspected gastroesophageal reflux or laryngopharyngeal reflux, proton pump inhibitors are essential for treatment.^[6]^ For patients with ineffective conservative treatment or high pathological classification, surgical intervention is recommended as soon as possible. Surgical procedures involve the use of cold instruments under different visual laryngoscopes or the application of carbon dioxide (CO_2_) laser therapy.

CO_2_ laser is a widely utilized and established medical laser technology in recent years, belonging to the mid-infrared range. It is a type of laser generated by molecular gas lasers, characterized by high brightness, high monochromaticity, and good coherence. It utilizes the vaporization, cutting, and coagulation properties of CO_2_ laser and is extensively employed in dermatology, reproductive system diseases, and other fields.^[7,8]^ Due to its precise removal of all visible lesions under microscopic visualization, it can mitigate complications caused by implantation, infection, instrument detachment, and traction, and results in minimal intraoperative bleeding and reduced damage to important laryngeal structures. Its application in otolaryngology has become increasingly prevalent and recommended by experts.^[9]^ However, there is a paucity of evidence-based support. The widespread adoption of CO_2_ laser technology in treating vocal cord leukoplakia has led to new findings and challenges. Some studies suggest that CO_2_ laser treatment may not significantly impact the progression of the condition and could potentially worsen postoperative voice quality.^[10,11]^ These findings highlight the need for further research and careful consideration of treatment options for patients with vocal cord leukoplakia.

To enhance the scientific evidence supporting CO_2_ laser treatment of vocal cord leukoplakia, we conducted a systematic review of relevant randomized controlled trial (RCT) studies, aiming to provide empirical support for clinical decision-making by physicians and patients.

## 2. Materials and methods

A meta-analysis was conducted to evaluate the efficacy and safety of CO_2_ laser treatment for vocal cord leukoplakia, adhering to the Preferred Reporting Items for Systematic Reviews and Meta-Analyses statement.^[12]^ The study was registered with PROSPERO under registration number CRD42024604109.

### 2.1. Search strategy

The database retrieval deadline for patients with vocal cord leucoplakia treated with carbon dioxide laser surgery was October 15, 2024. The search strategy employed keywords, titles, and full-text searches, including terms such as “vocal cord leukoplakia,” “laryngeal leukoplakia,” “precancerous lesions,” “laryngeal keratosis,” “benign vocal fold lesion,” “carbon dioxide laser therapy,” “CO_2_ laser,” “randomized clinical trials,” “clinical trials randomly,” and “RCTs.” The search encompassed 8 databases: PubMed, EMBASE, Web of Science, Cochrane, China National Knowledge Infrastructure, Chinese Science and Technology Periodical Database, Wangfang, and Chinese Biomedical Literature Database. All identified articles from the various databases were integrated into the article management software (EndNote X7) for subsequent analysis. For detailed information regarding specific search methodologies, please refer to the Supplementary File 1, Supplemental Digital Content, http://links.lww.com/MD/O382.

### 2.2. Inclusion criteria

Inclusion population: patients diagnosed with vocal cord leukoplakia and undergoing surgical treatment; Intervention group: patients undergoing CO_2_ laser surgery; Control group: patients undergoing non-CO_2_ laser surgery; RCT study; Primary outcomes: cure rate, recurrence rate, adverse reactions; Secondary outcome: vocal indicators.

### 2.3. Exclusion criteria

Nonrandomized controlled trials; inconsistent and missing data pre- and postintervention; redundant research; meta-analyses, case reports, editorials, academic papers, and animal studies.

### 2.4. Data collection

Data extraction will be conducted by Xingwei Cao and Yanan Zhou (independently) utilizing Microsoft Excel software through specific extraction tables. Discrepancies will be resolved by a third researcher Jiao Liang. Following full-text selection, the subsequent information will be extracted (if available): sample size, patient characteristics, diagnostic criteria, recurrence rate, recovery rate, complications, follow-up time, etc.

### 2.5. Quality assessment

The quality of the incorporated literature will be evaluated according to the methodological and quality standards delineated in the Cochrane Handbook for Systematic Reviews, emphasizing 7 key areas: randomization technique, allocation concealment, blinding procedures, outcome assessment bias, completeness of outcome data reporting, selective outcome reporting, and additional potential biases. Each of these aspects will be categorized as posing a “low risk,” “high risk,” or an “unclear risk” of bias, culminating in the compilation of a bias risk graph. This assessment will be conducted independently by 2 researchers, Xingwei Cao and Yanan Zhou, who will subsequently cross-verify their findings. In instances where discrepancies arise, a third researcher, Jiao Liang, will be consulted to collaboratively address and resolve the issues.

### 2.6. Strategy for data synthesis

Conduct a meta-analysis of the results utilizing Stata 18.0 and Revman 5.2 software (if applicable), encompassing cure rate, recurrence rate, adverse reactions, and vocal indicators. Generate visualized forest plots for these indicators. Employ *I*² to assess heterogeneity between studies, with values exceeding 50% indicating substantial heterogeneity. For datasets with *I*² > 50%, apply a random effects model for analysis; otherwise, utilize a fixed effects model and determine statistical significance with a *P* value threshold of <.05. In instances of significant heterogeneity, further investigation and examination of heterogeneity will be conducted using a one-by-one exclusion method. Additionally, funnel plots are employed to evaluate potential publication bias in the included studies.

## 3. Results

### 3.1. Search results

**Figure 1.**
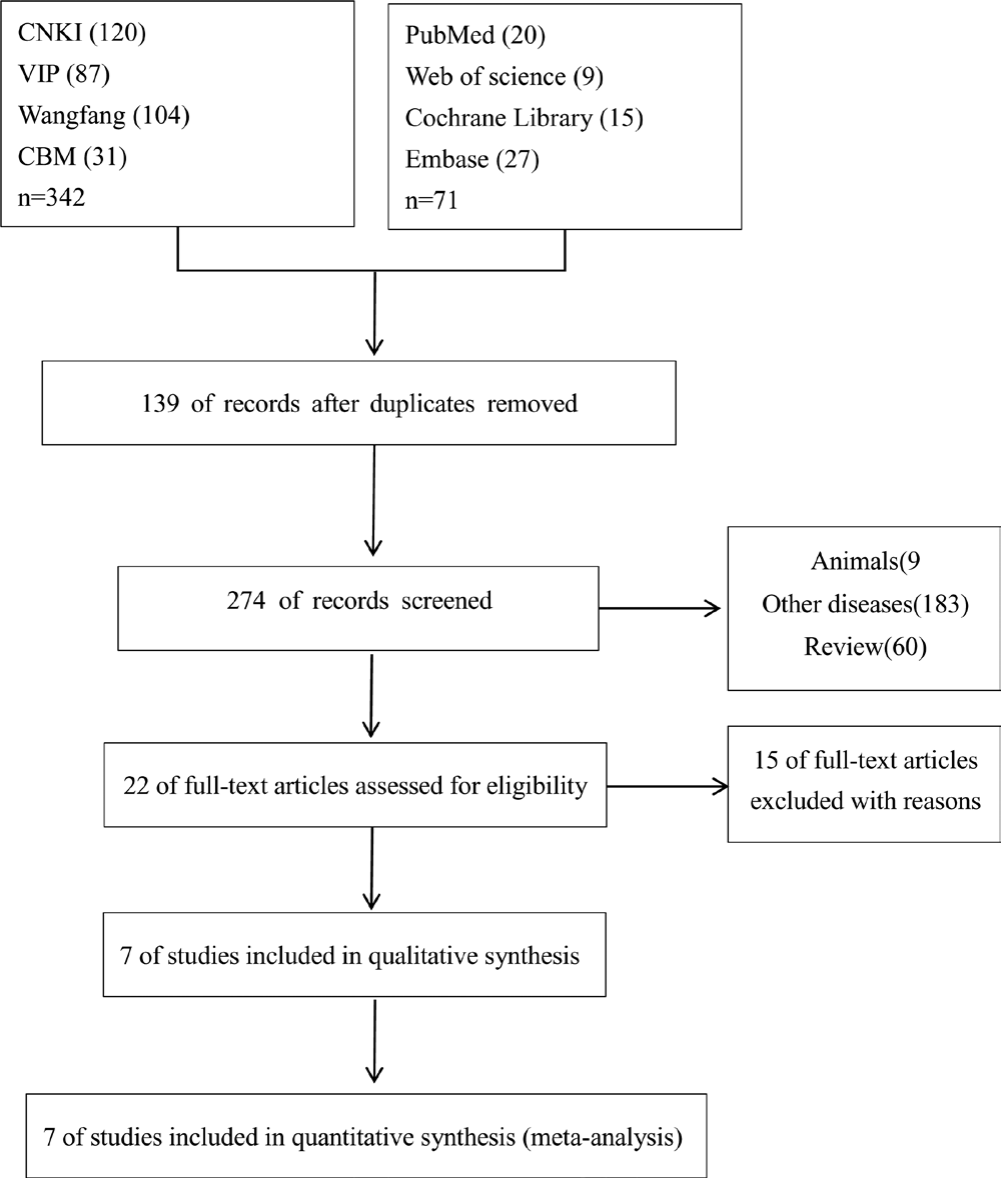
Flow diagram of the inclusion and exclusion process. CBM = Chinese Biomedical Literature Database, CNKI = China National Knowledge Infrastructure, VIP = Chinese Science and Technology Periodical Database.

An initial search of the database yielded 413 relevant studies. Utilizing EndNote X7, 139 duplicate studies were successfully eliminated. Following a comprehensive review of the titles and abstracts of the remaining 274 articles, 252 irrelevant articles were excluded, and the full texts of the remaining 22 articles were examined. Upon application of stringent inclusion and exclusion criteria, 7 articles were ultimately included in the meta-analysis. Figure 1 presents a detailed flowchart illustrating this process.

### 3.2. Study characteristic

The features incorporated in the study are delineated in Table 1. All included studies were randomized controlled trials with a minimum sample size of 40 and a maximum sample size of 100. The included studies were all conducted in China, with 1 case^[13]^ not specifying follow-up duration, 2 cases^[14,15]^ not documenting adverse reactions, 2 cases^[13,15]^ not specifying postoperative measures, 1 case^[15]^ not describing recovery, and 3 cases^[11,15,16]^ describing postoperative voice alterations.

**Table 1:** Basic characteristics of included literature.

Author year	Diagnostic criteria	Sample size		Male/female		Age (yr)		Course of disease		Vocal cords		Outcomes	Follow-up time	Adverse reactions	Postoperative measures
		E	C	E	C	E	C	E	C	E	C			E/C	
Zhao 2015	Pathological	15	15	22/8		29–76		1 mo–2 yr		Bilateral		①③	1–4 yr	NA	NA
Zhu 2005	NA	33	32	26/7	27/5	33–67	29–64	NA		Unilateral and bilateral		①②③	1–4 yr	NA	①②③④
Wu 2019	Pathological	20	20	13/7	12/8	29–72	27–70	3–21 mo	4–23 mo	NA		①②③④	1 yr	Tongue swelling/hoarseness = 1:0/1:1	①②③
Bao 2018	Pathological	43	43	35/8	37/6	32–67	34–68	2 mo–22 yr	1 mo–26 yr	NA		①②④	12 mo	No bleeding, vocal cord adhesion and vocal cord dyskinesia	②③
Wei 2018	Fibrolaryngoscope	40	40	23/27	24/26	26–74	26–74	2–18 yr	2–18 yr	U/B = 22/28 KWD(24) L1N1(15) L1N2(5) L1N3(6)	U/B = 21/29 KWD(25) L1N1(16) L1N2(5) L1N3(4)	①②④	NA	Cutting injury, bleeding,infection,others = 0:1:1:0/3:2:3:2	NA
Zhang 2018	Fibrolaryngoscope	39	39	32/7	33/6	30–69		2–24 mo		U/B = 31/8 KWD(22) L1N1(12) L1N2-L1N3(5)	U/B = 30/9 KWD(23) L1N1(10) L1N2-L1N3(6)	①②④	12–40 mo	Dyspnea, hemoptysis, vocal cord adhesion = 0:0:0/0:0:1	①③⑤
Gong 2015	Microscopic laryngoscope and strobe laryngoscope	30	30	27/3	26/4	31–74		2 mo–2 yr		U/B = 24/6 KWD(19) L1N1-L1N2(7) L1N3(4)	U/B = 25/5 KWD(17) L1N1-L1N2(9)L1N3(4)	①②③④	1 yr	Vocal cord adhesion = 0/1	①②③

B = bilateral, C = control group (routine operation), E = experiment group (CO_2_ laser surgery), KWD = keratosis without dysplasia, L1N1 = keratosis with mild dysplasia, L1N2 = keratosis with moderate dysplasia, L1N3 = keratosis with severe dysplasia or carcinoma in situ, NA = not mentioned, U = unilateral.

Outcomes: ① recurrence rate; ②efficiency; ③voice functions; ④adverse reactions.

Postoperative measures:①anti-infection; ②atomization; ③voice rest; ④ECG monitoring; ⑤breathing exercises.

### 3.3. Quality assessment of methodologies

**Figure 2.**
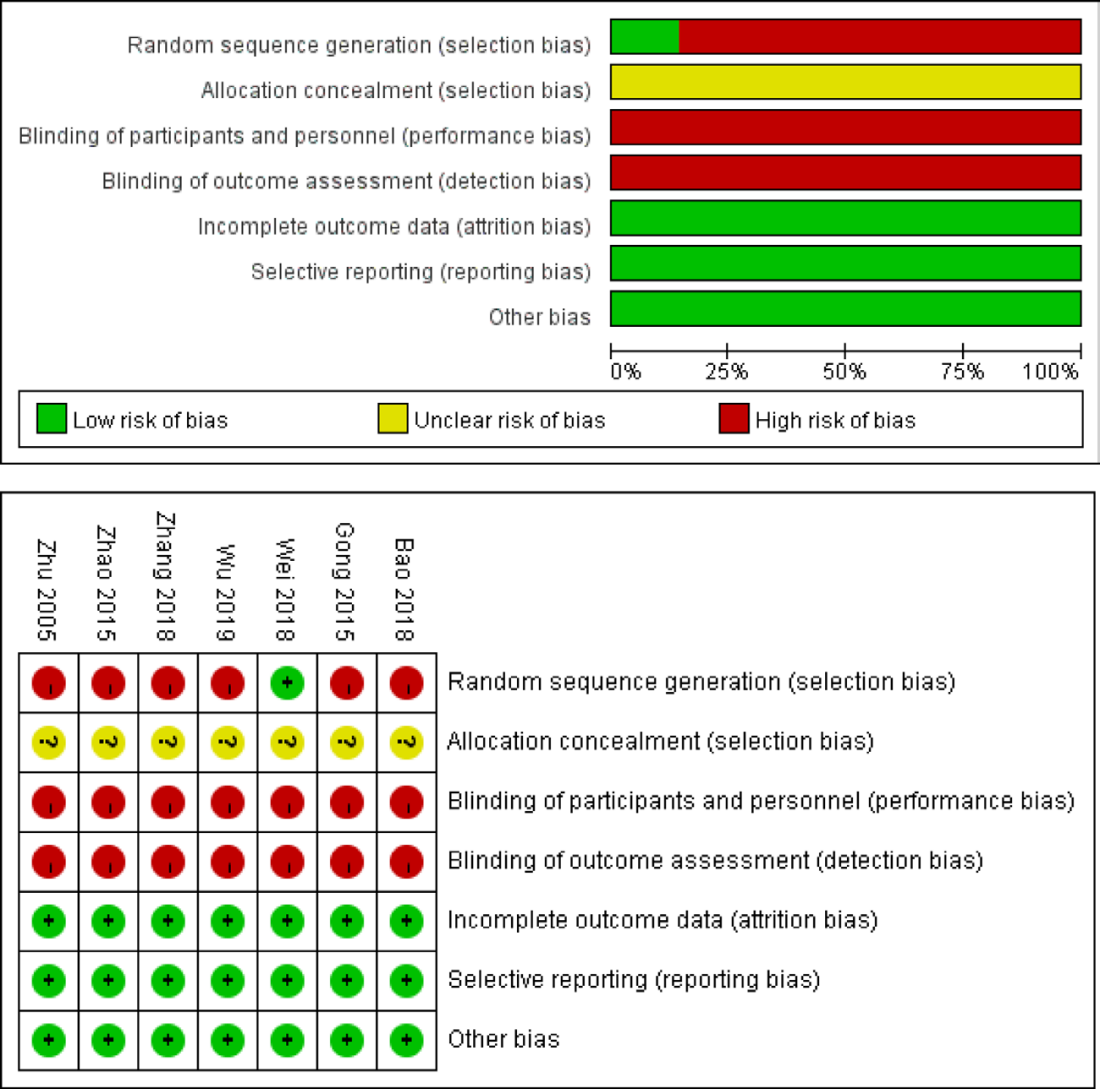
Summary of the risk of bias of the included studies.

The trials under examination provided comprehensive accounts of their randomization procedures.^[11,13,14,16–18]^ Notably, Wei^[13]^ utilized a random number table, whereas the remaining studies merely indicated the use of randomization without elaborating on specific techniques. Allocation concealment was not reported in any of the included studies. Given that the interventions involved distinct surgical treatments, blinding was not feasible, resulting in all trials being conducted without blinding. The research outcomes were reported comprehensively, with no evidence of selective reporting. Other potential biases may be present across all experiments. The risk of inclusion bias in the literature is illustrated in Figure 2.

### 3.4. Meta-analysis results

#### 3.4.1. Recurrence rate

**Figure 3.**
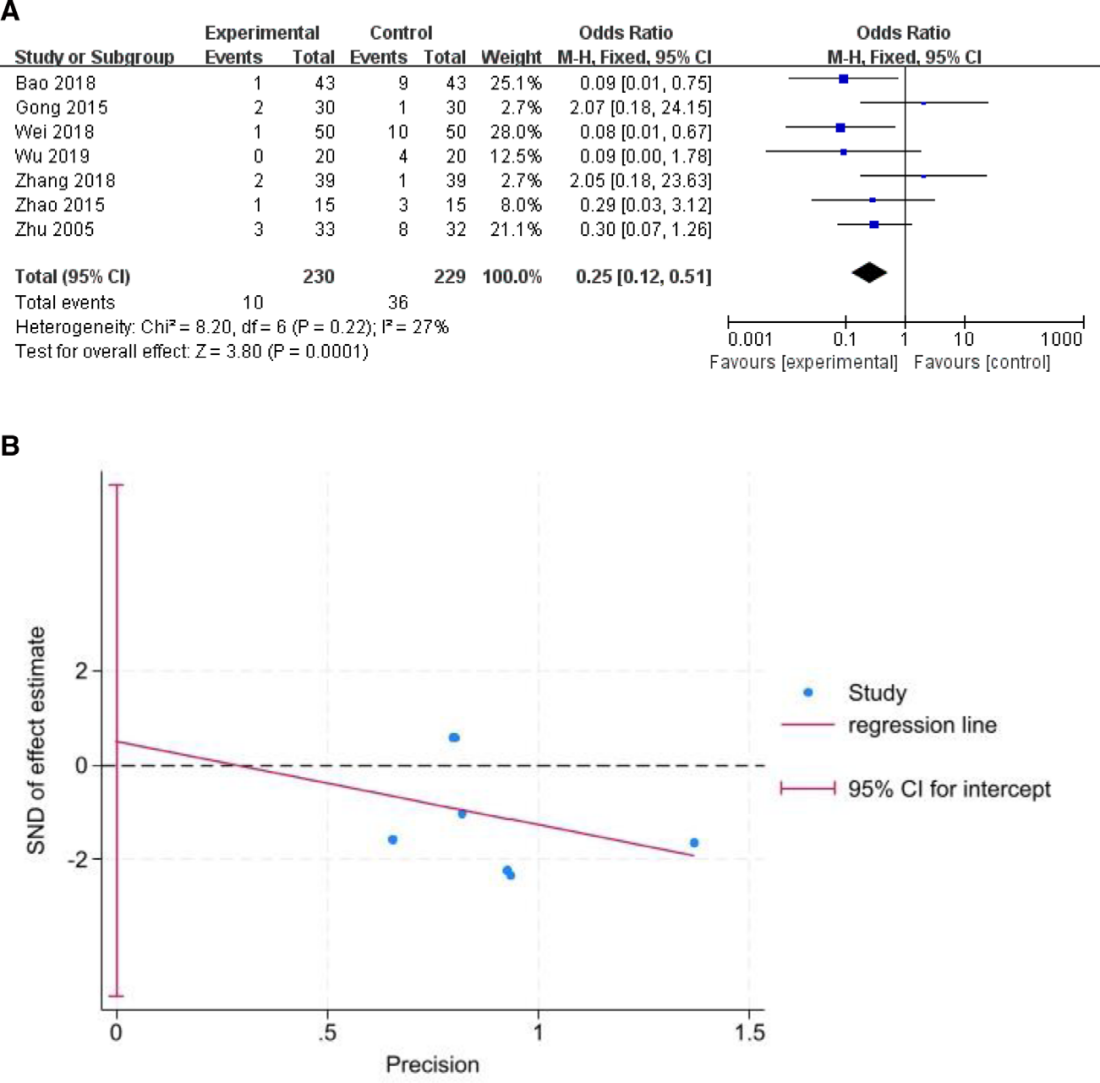
Results of the recurrence rate (A) and Egger test (B).

Seven trials encompassed 459 patients, with 230 in the CO_2_ laser group and 229 in the control group. The recurrence rate in the CO_2_ laser treatment group was significantly lower than that in the control group (odds ratio [OR] = 0.25, 95% confidence interval, CI [0.12, 0.51], *P* = .001; Fig. 3A). The Egger test (*P* = .815) indicated no significant publication bias (Fig. 3B).

Zhu et al,^[14]^ Zhao et al,^[15]^ and Zhang et al^[18]^ described the outcomes of patients with recurrence. Zhu et al^[14]^ reported that there were 8 cases of recurrence in the control group; 2 cases had no recurrence after CO_2_ laser treatment, 1 case underwent malignant transformation into cancer 2 years later and remains alive, and 5 patients underwent 3 surgeries without recurrence. Three patients in the laser group with recurrence underwent laser surgery again without recurrence. Zhao et al^[15]^ reported that among the 2 patients in the control group, 1 case did not recur after undergoing CO_2_ laser treatment again, and 1 case was lost to follow-up. In the CO_2_ laser group, 1 patient experienced recurrence and recovered after undergoing laser treatment again. Patients with recurrence in Zhang et al^[18]^ study experienced significant outcomes after undergoing 2 or more surgeries.

#### 3.4.2. Cure rate

**Figure 4.**
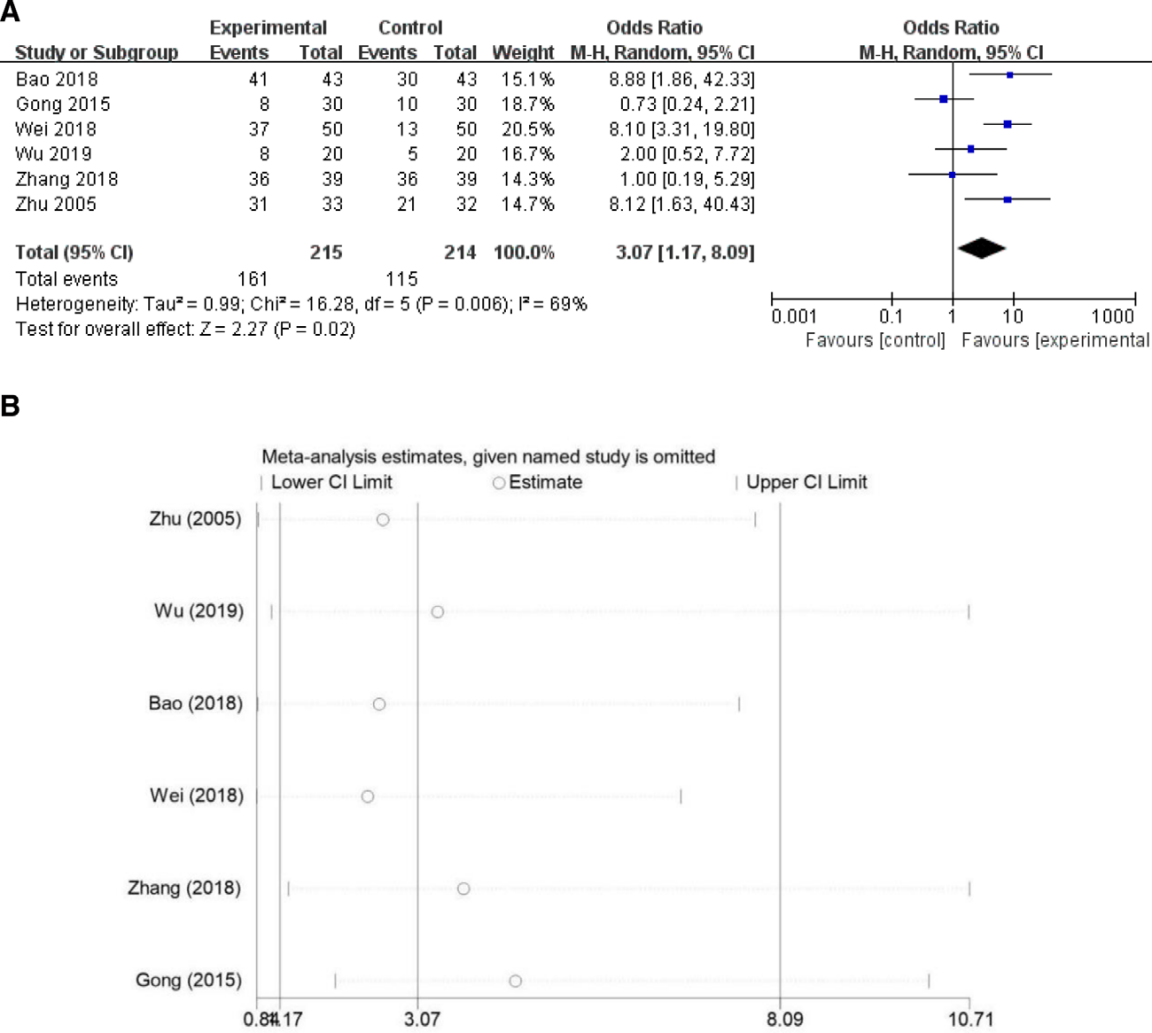
Results of the cure rate (A) and sensitivity analysis (B).

Six studies^[11,13,14,16–18]^ examined the cure rate, and due to heterogeneity (*I*^2^ = 69%, *P* = .006), a random-effects model was employed. The results indicated that the cure rate in the CO_2_ laser treatment group was significantly higher than in the control group (OR = 3.07, 95% CI [1.17, 8.09], *P* = .02; Fig. 4A). Sensitivity analysis was conducted utilizing a one-by-one exclusion method (Fig. 4B). The remaining combined results of the studies demonstrated no statistical significance and were consistent with the original combined results, suggesting that the results were robust.

#### 3.4.3. Changes in voice functions

**Figure 5.**
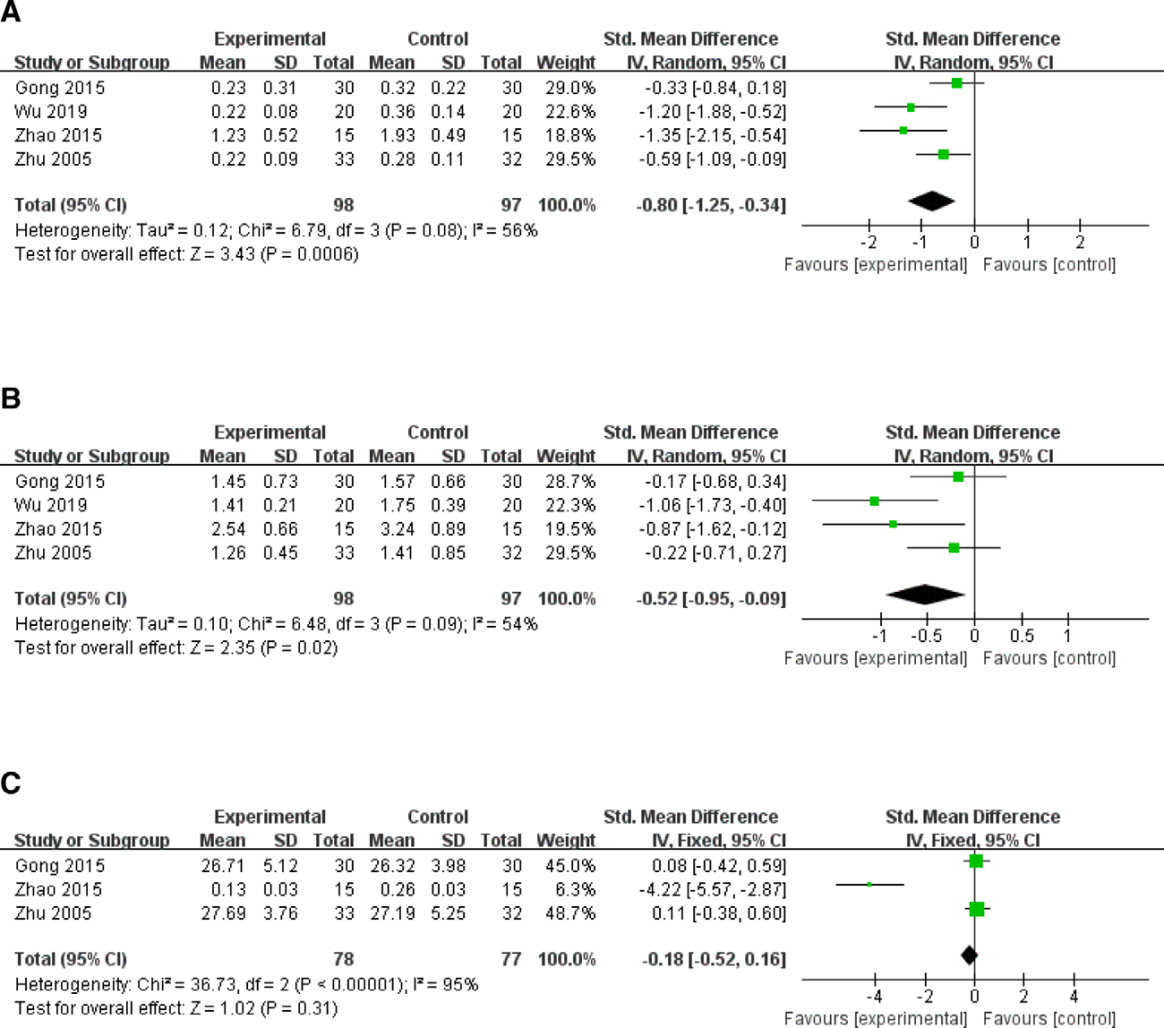
Results of the voice function, fundamental frequency disturbance (A), amplitude disturbance (B), signal-to-noise ratio (C).

Four studies^[11,14–16]^ examined the changes in voice function. A meta-analysis was conducted on the fundamental frequency disturbance, amplitude disturbance, and signal-to-noise ratio. Utilizing random effects analysis to evaluate the fundamental frequency disturbance (*P* = .08, *I*^2^ = 56%), the CO_2_ laser group demonstrated significantly lower values compared to the control group (SWD = −0.80, 95% CI [−1.25, −0.34], *P* = .0006; Fig. 5A). Employing a random effects model to assess amplitude perturbations (*P* = .09, *I*^2^ = 54%), the values in the CO_2_ laser group were significantly lower than those in the control group (SWD = −0.45, 95% CI [−0.74, −0.17], *P* = .002; Fig. 5B). Sensitivity analysis was conducted using a one-by-one exclusion method to demonstrate the robustness of the research results. Three studies investigated the harmonic noise ratio and analyzed it using random effects (*P* < .0001, *I*^2^ = 95%); however, there was no statistically significant difference between the 2 groups (SWD = −0.18, 95% CI [−0.52, 0.16], *P* = .31; Fig. 5C).

#### 3.4.4. Adverse reactions

**Figure 6.**
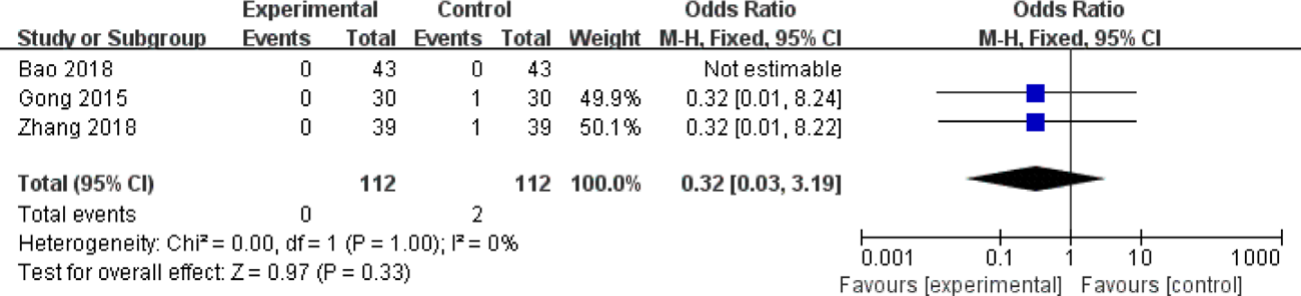
Results of the vocal cord adhesion.

Five studies^[11,13,16–18]^ reported adverse reactions, including tongue swelling,^[16]^ hoarseness,^[16]^ vocal cord adhesion,^[11,17,18]^ vocal cord movement disorders,^[17]^ cutting injuries,^[13]^ difficulty breathing,^[18]^ infection,^[13]^ bleeding,^[13,17]^ and coughing up bloody detcs.^[18]^ Meta analysis of vocal cord adhesion showed no significant difference between the 2 groups (OR = 0.32, 95% CI [0.03, 3.19], *P* = .33; Fig. 6).^[11,17,18]^ Overall, the incidence of adverse reactions in the CO_2_ laser treatment group was lower than that in the control group, but there was no significant difference between the 2 groups.

#### 3.4.5. Publication bias

**Figure 7.**
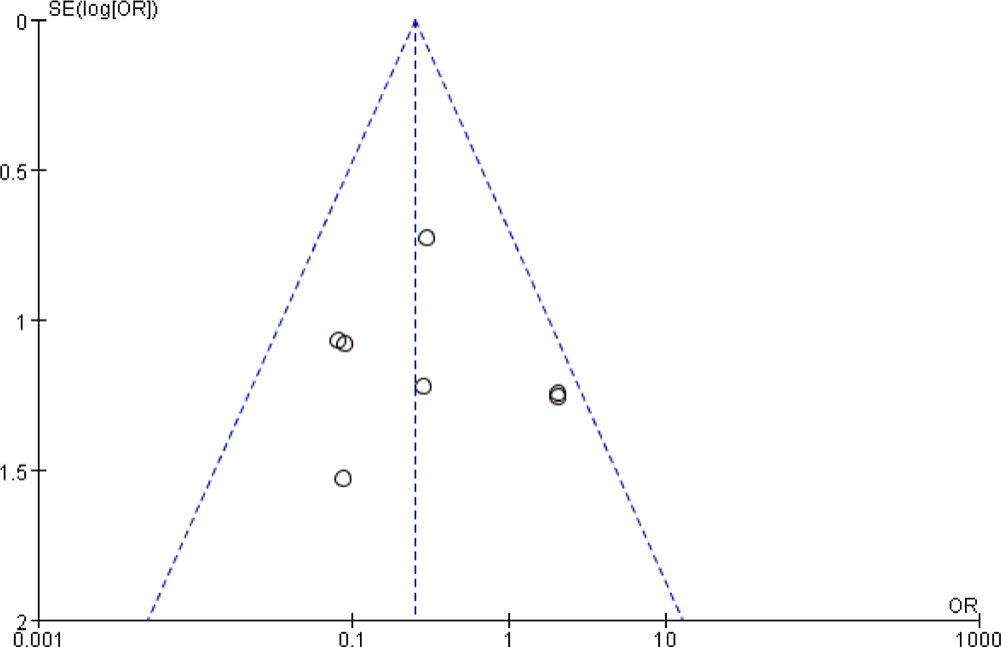
Funnel plot of publication bias.

The funnel plot was utilized to assess publication bias using the recurrence rate from 7 articles (Fig. 7). The plot exhibited symmetry, suggesting the absence of significant publication bias.

## 4. Discussion

Leukoplakia of the vocal cord is a keratoproliferative lesion of laryngeal mucosal epithelium. It is considered to be a precancerous lesion. Relevant studies have demonstrated that approximately 10% to 20% of leukoplakia cases with atypical hyperplasia progress to cancer, while more than half of the patients with severe dysplasia develop invasive cancer. The severity of dysplasia positively correlates with the rate of malignant transformation. In patients unresponsive to conservative treatment, early surgical resection of the lesion is recommended.^[11,19,20]^ CO_2_ laser resection is a novel surgical method that utilizes a support laryngoscope. This technique can directly vaporize small pathological tissue and seal dilated small blood vessels and lymphatic vessels on the vocal cord surface, potentially preventing the recurrence of vocal cord leukoplakia. Repeated surgeries are feasible in recurrent cases. This procedure offers advantages such as high precision, minimal damage, effective hemostasis, and clear surgical field visibility, garnering increasing attention in the field.^[21,22]^

Postoperative recurrence remains the primary concern in patients with vocal cord leukoplakia. This meta-analysis indicates that the recurrence rate in patients treated with CO_2_ laser is lower than that in patients treated with conventional surgery. Additionally, the cure rate was significantly higher in CO_2_ laser-treated patients than in those treated with conventional surgery. These findings are consistent with those of the previous studies.^[10,22]^ The literature has noted controversies regarding the prognosis of unilateral vs bilateral lesions and the different pathological types. However, the literature included in this study did not permit a combined analysis of these factors.

Recovery of voice function postsurgery is a crucial aspect of vocal cord lesion treatment. This meta-analysis suggests that CO_2_ laser surgery may offer certain advantages in the postoperative recovery of objective voice function, particularly in amplitude perturbation and fundamental frequency perturbation indices,^[23]^ these findings are consistent with those of the previous studies.^[10,24]^ However, additional studies are required to substantiate this conclusion.

Common adverse reactions across different surgical methods include swelling of the tongue. Although the overall incidence was lower in the CO_2_ laser treatment group than in the conventional surgery group, the difference was not statistically significant.

Wei^13]^ reported that the operation time for CO_2_ laser treatment was shorter than that of the conventional treatment group; however, relevant reports remain limited. In summary, CO_2_ laser treatment can reduce recurrence rates, improve cure rates, and enhance the postoperative voice quality. It represents a superior, minimally invasive procedure for vocal cord leukoplakia.

## 5. Limitations and suggestion for practice

This meta-analysis has several limitations. First, the primary literature originates from a single country, which may introduce significant bias. Second, the analysis does not account for the affected side of the vocal cord or pathological classification, potentially leading to result deviation. Third, substantial gender differences may exert an influence on the outcomes. Further large-sample studies are necessary, and it is advisable to analyze the affected vocal fold and various pathological classifications.

## 6. Conclusions and recommendations

The findings of this meta-analysis indicate that CO_2_ laser treatment significantly reduces the recurrence rate of vocal cord leukoplakia, enhances the cure rate, and contributes to the improvement of vocal function. CO_2_ laser treatment may be considered as a surgical approach worthy of further implementation in patients with vocal cord leukoplakia.

## Author contributions

**Data curation:** Yanan Zhou, Xingwei Cao.

**Methodology:** Jiao Liang.

**Project administration:** Xingwei Cao.

**Software:** Zhoujie Xu.

**Visualization:** Zhoujie Xu.

**Writing – original draft:** Yanan Zhou, Xingwei Cao.

**Writing – review & editing:** Jiao Liang.

## Supplementary Material


