# Supplementary material for: Safety and efficacy of carbon dioxide laser therapy for vocal cord leukoplakia: A systematic review and meta-analysis
Source: Medicine (Baltimore). 2025 Feb 14;104(7):e41539. doi: 10.1097/MD.0000000000041539 (PMC11835110; doi:10.1097/MD.0000000000041539)
Supplement: Supplementary file 1 [file medi-104-e41539-s001.docx]

**Full search strategy**

| **PubMed** |
| --- |
| ("RCT"[Title/Abstract] OR "rcts"[Title/Abstract] OR "randomized controlled trial"[Title/Abstract] OR "controlled clinical trial"[Title/Abstract] OR "randomized"[Title/Abstract] OR "clinical trials"[Title/Abstract]) AND (("lasers, gas"[MeSH Terms] OR ("lasers, gas"[MeSH Terms] OR ("lasers"[All Fields] AND "gas"[All Fields]) OR "gas lasers"[All Fields] OR ("carbon"[All Fields] AND "dioxide"[All Fields] AND "lasers"[All Fields]) OR "carbon dioxide lasers"[All Fields]) OR ("lasers, gas"[MeSH Terms] OR ("lasers"[All Fields] AND "gas"[All Fields]) OR "gas lasers"[All Fields] OR ("co2"[All Fields] AND "laser"[All Fields]) OR "co2 laser"[All Fields])) AND ("Leukoplakia"[MeSH Terms] OR "Vocal Cords"[MeSH Terms] OR (("larynges"[All Fields] OR "laryngitis"[MeSH Terms] OR "laryngitis"[All Fields] OR "laryngitides"[All Fields] OR "larynx"[MeSH Terms] OR "larynx"[All Fields] OR "laryngeal"[All Fields]) AND ("leucoplakias"[All Fields] OR "Leukoplakia"[MeSH Terms] OR "Leukoplakia"[All Fields] OR "leucoplakia"[All Fields] OR "leukoplakias"[All Fields])) OR (("Vocal Cords"[MeSH Terms] OR ("vocal"[All Fields] AND "cords"[All Fields]) OR "Vocal Cords"[All Fields]) AND ("leucoplakias"[All Fields] OR "Leukoplakia"[MeSH Terms] OR "Leukoplakia"[All Fields] OR "leucoplakia"[All Fields] OR "leukoplakias"[All Fields])) OR (("larynges"[All Fields] OR "laryngitis"[MeSH Terms] OR "laryngitis"[All Fields] OR "laryngitides"[All Fields] OR "larynx"[MeSH Terms] OR "larynx"[All Fields] OR "laryngeal"[All Fields]) AND "precancerous"[All Fields]) OR (("larynges"[All Fields] OR "laryngitis"[MeSH Terms] OR "laryngitis"[All Fields] OR "laryngitides"[All Fields] OR "larynx"[MeSH Terms] OR "larynx"[All Fields] OR "laryngeal"[All Fields]) AND ("keratose"[All Fields] OR "keratosic"[All Fields] OR "keratosis"[MeSH Terms] OR "keratosis"[All Fields] OR "keratoses"[All Fields])) OR (("larynges"[All Fields] OR "laryngitis"[MeSH Terms] OR "laryngitis"[All Fields] OR "laryngitides"[All Fields] OR "larynx"[MeSH Terms] OR "larynx"[All Fields] OR "laryngeal"[All Fields]) AND ("dysplasia"[All Fields] OR "dysplasias"[All Fields])) OR (("Vocal Cords"[MeSH Terms] OR ("vocal"[All Fields] AND "cords"[All Fields]) OR "Vocal Cords"[All Fields] OR ("vocal"[All Fields] AND "fold"[All Fields]) OR "vocal fold"[All Fields]) AND ("dysplasia"[All Fields] OR "dysplasias"[All Fields])) OR (("Vocal Cords"[MeSH Terms] OR ("vocal"[All Fields] AND "cords"[All Fields]) OR "Vocal Cords"[All Fields] OR ("vocal"[All Fields] AND "cord"[All Fields]) OR "vocal cord"[All Fields]) AND ("dysplasia"[All Fields] OR "dysplasias"[All Fields])))) |
| **Web of science** |
| #1 (TS=(vocal fold leukoplakia)) OR (QMTS=("benign vocal fold lesions")) OR (QMTS=("vocal fold leukoplakia")) OR (QMTS=("leukoplakia")) OR (QMTS=("laryngeal leukoplakia")) OR (QMTS=("benign vocal fold lesion")) OR (QMTS=("vocal cord leukoplakia")) OR (QMTS=("phonomicrosurgery")) OR (QMTS=("vocal fold")) OR (QMTS=("erythroplakia"))  #2 ((TS=(Carbon Dioxide Lasers)) OR TI=(Carbon Dioxide Lasers)) OR TI=(CO2 laser)  #3 #1 AND #2  #4 (((ALL=(RCT)) OR ALL=(randomized controlled trial)) OR ALL=(controlled clinical trial)) OR ALL=(clinical trials randomly)  #5 #3 AND #4 |
| **Cochrane** |
| #1 MeSH descriptor: [Vocal Cords] explode all trees  #2 MeSH descriptor: [Leukoplakia] explode all trees  #3 (laryngeal precancerous):ti,ab,kw OR (laryngeal keratosis):ti,ab,kw OR (laryngeal dysplasia):ti,ab,kw OR (vocal fold dysplasia):ti,ab,kw  #4 #1 OR #2 OR #3  #5 MeSH descriptor: [Lasers, Gas] explode all trees  #6 (Carbon Dioxide Lasers):ti,ab,kw OR (CO2 laser):ti,ab,kw  #7 #5 AND #6  #8 (RCT) OR (randomized clinical trials) OR (randomized controlled clinical trial) OR (randomized controlled trial) OR (randomized experiment)  #9 #8 AND #7 |
| **Embase** |
| 'vocal cord leukoplakia'/exp OR 'larynx disorder'/exp) AND 'carbon dioxide'/exp AND 'randomized controlled trial'/exp |
| **CNKI** |
| 篇名（声带白斑 + 声带白斑切除术 + 声带白斑手术 +喉白斑 + 喉白斑病 + 喉白斑疾病） +全文（癌前病变 + 癌前病变患者 + 癌前病变期 + 癌前病变组织 + 癌前病变阶段 + 癌前病变状态） AND 全文（二氧化碳激光 + 二氧化碳激光治疗 + 二氧化碳激光手术） AND 全文（随机对照 + 随机对照试验 + 随机对照研究 + '随机对照试验(rct)' + '随机对照试验(rcts)'）同义词扩展 |
| **VIP** |
| T=(声带白斑 OR 喉白斑 OR 癌前病变 OR 喉角化病 OR 早期喉癌） AND T=(二氧化碳激光 OR 手术 OR 二氧化碳 OR CO2） AND R=随机 |
| **Wangfang** |
| 题名或关键词:(声带白斑 or 喉白斑 or 喉角化病 or 癌前病变) and 题名或关键词:(二氧化碳激光 or 手术 or 激光) and 摘要:(随机 or 随机分配 or 随机对照试验 or 临床随机对照试验) |
| **CBM** |
| ((((("随机对照"[全部字段] OR ("随机对照试验"[全部字段] OR "Randomized Controlled Trial"[全部字段] OR "随机对照试验"[主题词]))) AND ((("二氧化碳激光"[标题] OR "气体激光"[标题] OR "Gas Lasers"[标题] OR "氮激光"[标题] OR "氙离子激光"[标题] OR "氦激光"[标题] OR "氦氖气体激光"[标题] OR "HeNe激光"[标题] OR "金属蒸气激光"[标题] OR "气体激光器"[标题] OR "氩离子激光"[标题] OR "CO2激光"[标题] OR "金蒸气激光"[标题]) OR ("CO2激光"[常用字段] OR "气体激光"[常用字段] OR "Gas Lasers"[常用字段] OR "氮激光"[常用字段] OR "氙离子激光"[常用字段] OR "氦激光"[常用字段] OR "氦氖气体激光"[常用字段] OR "HeNe激光"[常用字段] OR "氩离子激光"[常用字段] OR "二氧化碳激光"[常用字段]) OR "手术"[摘要])))) AND (("声带白斑"[标题] OR "喉白斑"[标题] OR "癌前病变"[全部字段] OR "喉角化病"[摘要]))) |
